# Estimating mortality attributable to alcohol or tobacco – a cohort study from Germany

**DOI:** 10.1186/s13011-025-00633-1

**Published:** 2025-01-22

**Authors:** Ulrich John, Hans-Jürgen Rumpf, Monika Hanke, Christian Meyer

**Affiliations:** 1 Dep Prevention Research and Social Medicine, University Medicine Greifswald, Institute of Community Medicine, W.-Rathenau-Str. 48, 17475 Greifswald, Germany; 2 Department of Psychiatry and Psychotherapy, Research Group S:TEP, University of Lübeck, Ratzeburger Allee 160, 23538 Lübeck, Germany

**Keywords:** Alcohol-attributable mortality, Tobacco-attributable mortality, Mortality cohort study, Risky alcohol consumption, Tobacco smoking

## Abstract

**Background:**

Little is known about mortality from four disorder combinations: fully attributable to alcohol or tobacco, partly attributable to both alcohol and tobacco, to tobacco only, to alcohol only.

**Aim:**

To analyze whether residents who had disclosed risky alcohol drinking or daily tobacco smoking had a shorter time to death than non-risky drinkers and never daily smokers twenty years later according to the disorder combinations.

**Methods:**

A random adult general population sample (4,075 study participants) of a northern German area had been interviewed in the years 1996–1997. Vital status and death certificate data were gathered 2017–2018. The data analysis included estimates of alcohol- or tobacco-attributable mortality using all conditions given in the death certificate and alternatively the underlying cause of death only.

**Results:**

Among 573 deaths, 71.9–94.1% had any alcohol- or tobacco-attributable disorder depending on the estimate. Risky alcohol consumption and daily tobacco smoking at baseline were related to disorders in the death certificate according to the combinations. Deaths with an alcohol- and tobacco-attributable disorder were related to risky alcohol consumption (subhazard ratio 1.57; 95% confidence interval 1.25–1.98) and to daily tobacco smoking at baseline (subhazard ratio 1.85; 95% confidence interval 1.42–2.41).

**Conclusion:**

First, more than 70% of the deceased persons had one or more alcohol- or tobacco-attributable disorders. This finding suggests that total mortality seems to be the suitable outcome if potential effects of alcohol or tobacco consumption in a general population are to be estimated. Second, the relations of risky alcohol consumption and tobacco smoking with time to death speak in favor of the validity of alcohol- and of tobacco-attributable disorders in death certificates and of considering both alcohol consumption and tobacco smoking if attributable deaths are to be estimated.

**Supplementary Information:**

The online version contains supplementary material available at 10.1186/s13011-025-00633-1.

## Background

Mortality attributable to alcohol or tobacco has been described to give evidence about deaths that are likely to be caused by alcohol consumption or tobacco smoking. Cohort study data revealed death to be earlier among consumers of higher compared to consumers of lower amounts of alcohol and among tobacco smokers compared to nonsmokers [1, 2]. The number of causes of death that are attributable to alcohol consumption or tobacco smoking increased by the years of research [3, 4], and 58 alcohol-attributable conditions among adults and children have been analyzed [5]. In a report of the Surgeon General in the United States of America, 21 causes of death have been established as attributable to tobacco smoking and estimated to be causally related to death [2]. An additional 14 disorders have been found as suggestive for a relation with tobacco smoking [6, 7]. In addition to attributable deaths [8], total mortality has been analyzed in relation to alcohol consumption and tobacco smoking [9].

Alcohol- or tobacco-attributable mortality data has been based on the underlying cause of death as documented in death certificates [1]. They are internationally standardized by the World Health Organization for routine use [10]. According to it, the underlying cause of death is the disease or injury that initiated the chain of disorders which led to death or the accident or violence that caused the fatal injury [10]. Further health disorders that are contributors to but not part of the fatal sequence of disorders leading to death are to be documented [10].

Death certificates have been reported to be biased by insufficiently valid entries about health disorders in the train of death. Studies detected more than 50% of death certificates to be faulty [11]. A comparison of death certificate with autopsy data in an institution in the United States of America suggests that 85% of the death certificates included one or more mistakes [12]. Three kinds of error are: missing or wrong underlying cause of death, wrong decision for one of the existing disorders as underlying cause of death, and wrong chains of disorders that led to death. A comparison of death certificates with medical records of the decedents in a hospital in the United States of America revealed that the underlying cause of death was false in 18% and in the wrong place in 26% of the death certificates [13]. False chains of disorders that led to death were found in 38% of death certificates in Spain [14]. Insufficiently specified disorders have been detected in 29% of the death certificates in an analysis in Norway [15]. This refers to disorders such as heart failure, sudden death, and exposure to an unspecified factor.

One means to reduce error might be to use all disorders that are part of the death certificate [16–20]. Reasons for this approach include the understanding that any certified health disorder may have added to death although its specific impact on initiating death is unknown. Health disorders may constitute a network which contributes to death. Furthermore, two or more underlying causes may exist [10]. According to an analysis in the United States of America, 75% of the certificates exhibited two or more causes of death [20]. The utilization of all disorders instead of one underlying cause only has the potential to provide more information about existing alcohol- or tobacco-attributable health disorders. Using all disorders reported in the death certificate, alcohol-attributable and established plus suggestive tobacco-attributable disorders may be estimated or alternatively established but not suggestive tobacco-attributable disorders. Two further estimates are restricted to underlying causes of death and established plus suggestive tobacco-attributable disorders or alternatively established but not suggestive tobacco-attributable disorders. Little is known about these four estimates in one analysis.

Alcohol- or tobacco-attributable health disorders appear in four combinations: fully attributable to alcohol or tobacco, partly attributable to both alcohol and tobacco, to tobacco only, or to alcohol only. Fully attributable are those disorders that by definition may be present only in persons who had drunk alcohol or smoked tobacco. Partly attributable are disorders that exist in a larger proportion of high compared to low alcohol consumers or tobacco smokers compared to never smokers. These diseases or injuries may be caused by alcohol, tobacco or other factors. Fifteen health disorders have been fully and another 43 partly attributed to alcohol consumption [1]. In the United States of America, 71% of the alcohol-attributable deaths in the year 2020–2021 were partially attributable to alcohol consumption [5]. Thirty-five health disorders or groups of disorders have been attributed to tobacco smoking [6, 7]. Among them, 21 are established as causally linked to tobacco smoking [2] and an additional 14 as suggestive for a relation with tobacco smoking [6, 7]. According to evidence, increased mortality exists for established and for suggestive disorders [7]. Attributable mortality has mostly been estimated for risky alcohol drinking or for tobacco smoking alone although these two risk behaviors may have a combined impact on mortality risk [21–23]. The contribution of one alcoholic drink was found to be equal to one cigarette [21].

Two limitations of the evidence so far are: First, little is known about the prevalence of alcohol- or tobacco-attributable disorders according to the four estimates and the four combinations. Second, we lack knowledge about how strongly risky alcohol consumption and tobacco smoking are related to death according to the estimates and the combinations. Time to death may be assumed to be dependent on the four combinations of risk behavior. Evidence according to this is lacking although it is important for the provision of evidence about potential causes of death and for the prevention of alcohol- or tobacco-related disorders. Findings may inform preventive efforts to possibly address alcohol drinking and tobacco smoking together instead of each alone.

The purpose of this study was, first, to estimate the prevalence of the four combinations of alcohol- or tobacco-attributable disorders using the four estimates. Second, we wanted to provide evidence about the validity of disorders in the death certificate. We analyzed whether risky alcohol drinking and daily tobacco smoking in the past are related to time to death in accordance with the four combinations using the four estimates.

## Methods

### Sample

A random adult general population sample had been drawn from a total of 193,452 residents at age 18 to 64 in a northern German area [24] and baseline interviews were completed from July 20,1996, to March 18, 1997. The sample was representative for the region under study. Among the 5,829 eligible residents, 1754 (30.1%) could not be reached, refused the interview, were deceased or mentally severely handicapped, and 4,075 (69.9%) provided interview data which were analyzed [24]. For the 4,075 study participants at baseline, we assessed vital status from April 1, 2017, to April 30, 2018. The median time from baseline to mortality follow-up was 20.6 years [25].

### Assessments at baseline

The quantity and frequency of alcohol consumption had been assessed as part of the internationally standardized Munich-Composite International Diagnostic Interview [26]. Risky alcohol drinking was assumed if the study participant had drunk 20 or more grams pure alcohol per day (females) or 40 or more grams per day (males), harmful consumption if females had drunk 40 or more and males 60 or more grams pure alcohol per day in the last twelve months or any time period of 6 or more months in the time before the last twelve months prior to the interview. Tobacco smoking status was determined by questions according to smoking in life before. Never smokers were those who answered “No” to the question whether they ever had smoked tobacco by cigarette, cigar or pipe in their life before. Never daily smokers reported a history of smoking but not daily over a time period longer than 4 weeks in their life before. Former daily smokers had smoked daily longer than four weeks in the past but not in the last twelve months prior to the interview. Current daily smokers had smoked tobacco daily in the last twelve months prior to the interview.

### Assessments at mortality follow-up

Residents’ registration offices of the last place of residence provided vital status data. For deceased persons, death certificates and autopsy reports were given by the health authorities of the community where the individual had died. The death certificate information was delivered on paper by forms in accordance with the World Health Organization [10]. For death certification or autopsy reporting, disorders could be entered in four data fields: immediately leading to death, antecedent disorder (disorder that led to the disorder which immediately led to death), underlying causes of death, and further disorders. According to the death certificate form, further disorders are disorders that have contributed to death without being related to the cause of death [10]. If no underlying cause of death existed in the death certificate the antecedent cause, and if this had not been provided also, the disorder that immediately led to death was assumed as underlying cause of death [10].

### Data analysis

We used the alcohol-attributable chronic or acute disorders for adults published by the Centers for Disease Control in the United States of America [1, 5] and the tobacco-attributable disorders for which a causal relation with tobacco smoking has been referred to as established [2, 7]. Also, additional disorders that have been revealed by data to be related to tobacco smoking were included. These disorders are suggestive for a relation with tobacco smoking [6, 7]. Disorders may be fully or partly attributable. The fully attributable included diseases or injuries attributable to alcohol [1]. We added mental and behavioral disorders due to tobacco use or due to psychoactive substance use other than alcohol or nicotine to the fully alcohol-attributable disorders.

We used all disorders of both the death certificate and the autopsy data and analyzed four estimates and four combinations of alcohol- or tobacco-attributable disorders in the death certificates. The first estimate included the alcohol-attributable and the established and suggestive tobacco-attributable among all disorders of the death certificate, the second estimate the alcohol-attributable and established tobacco-attributable disorders only among all disorders in the death certificate. The third estimate was restricted to the alcohol-attributable disorders and the established and suggestive tobacco-attributable among the underlying causes of death only, and the fourth estimate was restricted to the alcohol-attributable disorders and the established tobacco-attributable only among the underlying causes of death.

For building the combinations of attributable disorders each disorder in the death certificate was allocated to one of seven mutually exclusive categories on grounds of the International Classification of Diseases, version 10, of the World Health Organization [27]: fully attributable, partly attributable to alcohol and tobacco (established tobacco-attributable disorders), partly attributable to alcohol and tobacco (suggestive tobacco-attributable disorders), partly attributable to tobacco only (established tobacco-attributable disorders), partly attributable to tobacco only (suggestive tobacco-attributable disorders), and partly attributable to alcohol only (Additional file 1). All other disorders were not attributable.

The four combinations of attributable disorders were: fully attributable disorders, partly alcohol- and tobacco-attributable, partly tobacco-attributable only, and partly alcohol-attributable only. The four combinations were calculated in the same hierarchical order for all estimates. In a first step, we estimated the fully attributable disorders. Second, among the remaining deaths the partly alcohol- and tobacco-attributable, third, among the then remaining deaths disorders partly attributable to tobacco only, and among the then remaining deaths, disorders partly attributable to alcohol only were determined. The deaths which had no attributable disorder at all were the group of not attributable disorder. Each disorder in the death certificate and in the autopsy report was coded according to the International Classification of Diseases, version 10, of the World Health Organization [27].

According to the second aim, we assumed that increased mortality would be found in the combinations of the alcohol- and tobacco-attributable deaths: (1) Disorders that are fully attributable or partly attributable to alcohol and to tobacco should be more likely both among risky than among non-risky alcohol consumers and among daily than among never or never daily smokers. (2) Disorders partly attributable to smoking only should be more likely among daily tobacco smokers but not among risky alcohol consumers. (3) Disorders partly attributable to alcohol consumption only should be more likely among risky alcohol drinkers but not among daily tobacco smokers. (4) Disorders that are not attributable should not be more likely among risky alcohol consumers and among daily tobacco smokers than among non-risky alcohol drinkers and among nonsmokers respectively.

The proportions that are presented refer to all deaths. We analyzed lifetime from baseline to mortality follow-up or death dependent on risky alcohol consumption and tobacco smoking and used competitive-risk regression [28]. Competitive-risk regression allows to consider competing deaths from other combinations when analyzing the deaths in one combination. The probability of death in one combination is altered by deaths in the other combinations. We present the subhazard ratio (SHR) and 95% confidence interval (CI) adjusted for age and sex. Reference groups were study participants who had been non-risky drinkers or never or never daily smokers before baseline. We considered subhazard ratios with both boundaries of the confidence interval being 1 or higher as significantly increased. We analyzed groups of five or more deaths per predictor variable [29]. If this precondition was not fulfilled no subhazard is given. The data analysis was performed using the Stata 18 software [30].

## Results

Among the 4,075 study participants, for 4,028 (98.8%) vital status data were received, and we found 573 deaths. Among the 2,006 female study participants with vital status information, 222 (11.1%), among the 2,022 male participants with vital status information, 351 (17.4%) had been dead. For 5.2% of the 573 decedents, no information from death certificates or autopsy reports was available, and data about health disorders were found in 543 death certificates. The mean number of 4.48 disorders had been declared in the death certificates and autopsy reports taken together (standard deviation 2.11; median 4, range 1 to 11). The maximum number of disorders entered in the death certificate or autopsy report was: 2 disorders immediately leading to death, 3 antecedent disorders, 3 underlying causes of death, and 7 further disorders. The underlying cause of death field had entries in 396 (72.9%) of the death certificates and autopsy reports taken together. Among these entries, the mean number of diagnoses was 1.25 (standard deviation 0.58; median 1). For the remaining 147 (27.1%) death certificates, the underlying cause of death had to be substituted by the disorder that immediately led to death or the antecedent disorder.

**Table 1:** Alcohol- or tobacco-attributable disorders in death certificates

Death certificate: estimates of disorders	Death certificate: disorder combinations attributable to									Disorder not attributable	Death certificate unavailable	Death cases total
	alcohol or tobacco fully		alcohol + tobacco partly		tobacco only partly		alcohol only partly		alcohol or tobacco total			
	*N* %		*N* %		*N* %		*N* %		*N* %	*N* %	*N* %	*N* %
All disorders, including established and suggestive tobacco-attributable	69 12.0		343 59.9		105 18.3		22 3.8		539 94.1	4 0.7	30 5.2	573 100.0
All disorders, including established tobacco-attributable only	69 12.0		297 51.8		76 13.3		68 11.9		510 89.0	33 5.8	30 5.2	573 100.0
Underlying cause of death, including established and suggestive tobacco-attributable	26 4.5		210 36.6		229 40.0		21 3.7		486 84.8	57 9.9	30 5.2	573 100.0
Underlying cause of death, including established tobacco-attributable only	26 4.5		163 28.4		155 27.1		68 11.9		412 71.9	131 22.9	30 5.2	573 100.0

All disorders: all disorders mentioned in death certificate

100.0% may include rounding errors

Any alcohol- or tobacco-attributable disorder was present, depending on the estimate, in 71.9 to 94.1% of the deaths or 75.9 to 99.3% of the death certificates that had been available (Table 1). If all conditions of the death certificates were considered disorders fully attributable to alcohol or tobacco existed for 12.0% of the decedents. Among these, for 73.9% the disorder was alcohol-attributable. Disorders partly attributable both to alcohol and to tobacco were revealed by the data for 59.9% of all deaths if all disorders and established plus suggestive tobacco-attributable disorders were taken into account. Mental disorders due to psychoactive substance use other than alcohol drinking or tobacco smoking did not provide additional attributable deaths.

**Table 2:** Alcohol consumption and tobacco smoking at baseline and alcohol- or tobacco-attributable diagnoses in death certificates

Baseline: alcohol drinking or tobacco smoking	Death certificate: disorder combinations attributable to										Death certificate: disorder not attributable		Death certificate unavailable		Death cases total
	alcohol or tobacco fully		alcohol + tobacco partly		tobacco only partly		alcohol only partly		alcohol or tobacco total						Total
	*N* %		*N* %		*N* %		*N* %		*N* %		*N* %		*N* %		*N* %
	**Estimate: All disorders**,** including established and suggestive tobacco-attributable**														
Alcohol risk drinking															
No risk drinking	39 10.2		226 58.9		78 20.3		16 4.2		359 93.5		3 0.8		22 5.7		384 100.0
Risk drinking	30 15.9		117 61.9		27 14.3		6 3.2		180 95.2		1 0.5		8 4.2		189 100.0
Total	69 12.0		343 59.9		105 18.3		22 3.8		539 94.1		4 0.7		30 5.2		573 100.0
Tobacco smoking															
Never or never daily	9 5.5		104 63.8		29 17.8		8 4.9		150 92.0		4 2.5		9 5.5		163 100.0
Former daily	13 10.8		86 71.7		8 6.7		7 5.8		114 95.0		0 0.0		6 5.0		120 100.0
Current daily	47 16.2		153 52.8		68 23.4		7 2.4		275 94.8		0 0.0		15 5.2		290 100.0
Total	69 12.0		343 59.9		105 18.3		22 3.8		539 94.1		4 0.7		30 5.2		573 100.0
	**Estimate: All disorders**,** including established tobacco-attributable only**														
Alcohol risk drinking															
No risk drinking	39 10.2		196 51.0		56 14.6		46 12.0		337 87.8		25 6.5		22 5.7		384 100.0
Risk drinking	30 15.9		101 53.4		20 10.6		22 11.6		173 91.5		8 4.2		8 4.2		189 100.0
Total	69 12.0		297 51.8		76 13.3		68 11.9		510 89.0		33 5.8		30 5.2		573 100.0
Tobacco smoking															
Never or never daily	9 5.5		83 50.9		18 11.0		29 17.8		139 85.3		15 9.2		9 5.5		163 100.0
Former daily	13 10.8		80 66.7		6 5.0		13 10.8		112 93.3		2 1.7		6 5.0		120 100.0
Current daily	47 16.2		134 46.2		52 17.9		26 9.0		259 89.3		16 5.5		15 5.2		290 100.0
Total	69 12.0		297 51.8		76 13.3		68 11.9		510 89.0		33 5.8		30 5.2		573 100.0
	**Estimate: Underlying cause of death**,** including established and suggestive tobacco-attributable**														
Alcohol risk drinking															
No risk drinking	15 3.9		137 35.7		148 38.5		15 3.9		315 82.0		47 12.2		22 5.7		384 100.0
Risk drinking	11 5.8		73 38.6		81 42.9		6 3.12		171 90.5		10 5.3		8 4.2		189 100.0
Total	26 4.5		210 36.6		229 40.0		21 3.7		486 84.8		57 9.9		30 5.2		573 100.0
Tobacco smoking															
Never or never daily	3 1.8		65 39.9		53 32.5		9 5.5		130 79.8		24 14.7		9 5.5		163 100.0
Former daily	4 3.3		57 47.5		34 28.3		5 4.2		100 83.3		14 11.7		6 5.0		120 100.0
Current daily	19 6.6		88 30.3		142 49.0		7 2.4		256 88.3		19 6.6		15 5.2		290 100.0
Total	26 4.5		210 36.6		229 40.0		21 3.7		486 84.8		57 9.9		30 5.2		573 100.0
	**Estimate: Underlying cause of death**,** including established tobacco-attributable only**														
Alcohol risk															
No risk drinking	15 3.9		113 29.4		99 25.8		39 10.2		266 69.3		96 25.0		22 5.7		384 100.0
Risk drinking	11 5.8		50 26.5		56 29.6		29 15.3		146 77.2		35 18.5		8 4.2		189 100.0
Total	26 4.5		163 28.4		155 27.1		68 11.9		412 71.9		131 22.9		30 5.2		573 100.0
Tobacco smoking															
Never or never daily	3 1.8		44 27.0		32 19.6		30 18.4		109 66.9		45 27.6		9 5.5		163 100.0
Former daily	4 3.3		46 38.3		25 20.8		16 13.3		91 75.8		23 19.2		6 5.0		120 100.0
Current daily	19 6.6		73 25.2		98 33.8		22 7.6		212 73.1		63 21.7		15 5.2		290 100.0
Total	26 4.5		163 28.4		155 27.1		68 11.9		412 71.9		131 22.9		30 5.2		573 100.0

All disorders: all disorders mentioned in death certificate

Risk drinking: females more than 20 g, males more than 40 g pure alcohol per day

Former daily: smoked daily before but not within the last twelve months

Current daily: smoked daily within the last twelve months

100.0% may include rounding errors

**Table 3:** Alcohol consumption and tobacco smoking at baseline and alcohol- or tobacco-attributable diagnoses in death certificates

Baseline: alcohol drinking or tobacco smoking	Death certificate: disorder combinations attributable to										Death certificate: disorder not attributable		Death certificate unavailable
	alcohol or tobacco fully		alcohol + tobacco partly		tobacco only partly		alcohol only partly		alcohol or tobacco total				
	SHR CI		SHR CI		SHR CI		SHR CI		SHR CI		SHR CI		SHR CI
	**Estimate: All disorders**,** including established and suggestive tobacco-attributable**												
	**Death cases**: **69**		**Death cases: 343**		**Death cases: 105**		**Death cases**: **22**		**Death cases: 539**		**Death cases**: **4**		**Death cases**: **30**
Alcohol risk drinking													
No risk drinking	reference		reference		reference		reference		reference				reference
Risk drinking	2.05 1.25–3.36		1.57 1.25–1.98		1.00 0.62–1.60		1.39 0.50–3.87		1.56 1.30–1.88		-		1.15 0.53–2.51
Tobacco smoking													
Never or never daily	reference		reference		reference		reference		reference				reference
Former daily	1.73 0.73–4.08		1.11 0.82–1.51		0.42 0.19–0.93		1.26 0.44–3.63		1.03 0.80–1.33		-		1.00 0.37–2.73
Current daily	4.71 2.27–9.76		1.85 1.42–2.41		2.96 1.86–4.72		0.75 0.24–2.30		2.33 1.89–2.88		-		1.85 0.85–4.06
	**Estimate: All disorders**,** including established tobacco-attributable only**												
	**Death cases**: **69**		**Death cases: 297**		**Death cases**: **76**		**Death cases**: **68**		**Death cases**: **510**		**Death cases**: **33**		**Death cases**: **30**
Alcohol risk drinking													
No risk drinking	reference		reference		reference		reference		reference		reference		reference
Risk drinking	2.05 1.25–3.36		1.48 1.16–1.90		0.98 0.57–1.67		1.84 1.09–3.11		1.59 1.31–1.91		1.16 0.47–2.84		1.15 0.53–2.51
Tobacco smoking													
Never or never daily	reference		reference		reference		reference		reference		reference		reference
Former daily	1.73 0.73–4.08		1.26 0.91–1.74		0.53 0.21–1.36		0.67 0.33–1.34		1.10 0.85–1.42		-		1.00 0.37–2.73
Current daily	4.71 2.27–9.76		2.03 1.52–2.71		4.00 2.30–6.94		0.91 0.52–1.59		2.39 1.92–2.96		1.11 0.51–2.41		1.85 0.85–4.06
	**Estimate: Underlying cause of death**,** including established and suggestive tobacco-attributable**												
	**Death cases**: **26**		**Death cases: 210**		**Death cases**: **229**		**Death cases**: **21**		**Death cases**: **486**		**Death cases**: **57**		**Death cases**: **30**
Alcohol risk drinking													
No risk drinking	reference		reference		reference		reference		reference		reference		reference
Risk drinking	2.00 0.91–4.39		1.59 1.18–2.13		1.52 1.14–2.03		1.56 0.57–4.26		1.67 1.38–2.02		0.72 0.35–1.47		1.15 0.53–2.51
Tobacco smoking													
Never or never daily	-		reference		reference		reference		reference		reference		reference
Former daily	-		1.15 0.79–1.66		0.86 0.55–1.33		0.78 0.24–2.58		1.02 0.78–1.34		0.95 0.47–1.91		1.00 0.37–2.73
Current daily	-		1.56 1.12–2.17		3.27 2.32–4.60		0.68 0.23–2.02		2.44 1.95–3.05		1.03 0.56–1.90		1.85 0.85–4.06
	**Estimate: Underlying cause of death**,** including established tobacco-attributable only**												
	**Death cases**: **26**		**Death cases: 163**		**Death cases**: **155**		**Death cases**: **68**		**Death cases**: **412**		**Death cases**: **131**		**Death cases**: **30**
Alcohol risk drinking													
No risk drinking	reference		reference		reference		reference		reference		reference		reference
Risk drinking	2.00 0.91–4.39		1.24 0.88–1.74		1.50 1.06–2.11		2.73 1.65–4.51		1.66 1.35–2.04		1.16 0.76–1.75		1.15 0.53–2.51
Tobacco smoking													
Never or never daily	-		reference		reference		reference		reference		reference		reference
Former daily	-		1.35 0.89–2.05		1.03 0.60–1.76		0.69 0.35–1.36		1.10 0.83–1.47		0.76 0.45–1.29		1.00 0.37–2.73
Current daily	-		1.89 1.30–2.76		3.78 2.44–5.86		0.73 0.40–1.33		2.38 1.86–3.04		1.65 1.11–2.45		1.85 0.85–4.06

SHR: Subhazard ratio

CI: 95-%-confidence interval

All disorders: all disorders mentioned in death certificate

Risk drinking: females more than 20 g, males more than 40 g pure alcohol per day

Former daily: smoked daily before but not within the last twelve months

Current daily: smoked daily within the last twelve months

- no result presented due to number of death cases less than 5

Of the deceased persons with risky alcohol consumption at baseline, any disorder in the death certificate that is fully attributable to alcohol or tobacco was found for 15.9% (Table 2). Among these, 29.0% had a harmful alcohol consumption in their lifetime before baseline compared to 11.3% among persons with any partly attributable disorder. The risky alcohol drinkers at baseline included 61.9% with a disorder that is partly attributable to alcohol and to tobacco as well if established and suggestive tobacco-attributable disorders were considered. Among the current daily smokers at baseline who were deceased, 16.2% had any fully attributable disorder in the death certificate and 52.8% any disorder that is attributable to alcohol consumption and to tobacco smoking whenever the estimate included established and suggestive tobacco-attributable disorders.

For deceased persons who at baseline had drunk alcohol in a risky manner or smoked tobacco daily, the data of all four estimates revealed significantly increased subhazard ratios (Table 3). The estimates that include all disorders revealed significantly increased subhazard ratios for disorders that are fully attributable and disorders that are partly attributable both to alcohol and to tobacco. The subhazard ratio of those with risky drinking at baseline was 2.05 (1.25–3.36) in the group of deceased persons with a fully attributable disorder and 1.48 (1.16–1.90) in the group of deceased persons with partly alcohol- and tobacco-attributable disorders if all disorders in the death certificate are considered including established but not suggestive tobacco-attributable. Current daily smokers had a subhazard ratio 4.71 (2.27–9.76) for fully attributable disorders and 2.03 (1.52–2.71) for partly alcohol- and tobacco-attributable disorders if among the tobacco-attributable only established ones are considered. The four assumptions according to the combinations of alcohol- or tobacco-attributable disorders were fulfilled when all disorders and the established tobacco-attributable disorders only but not when the established and suggestive tobacco-attributable disorders were used. With respect to the underlying causes of death, one of the four assumptions was fulfilled, both when established and suggestive or when established tobacco-attributable disorders only were used. Former daily smoking at baseline was not related to time to death in any of the estimates. According to disorders which were neither attributable to alcohol nor to tobacco, no significantly increased relation was present except for established tobacco-attributable disorders with current daily smoking as the predictor.

## Discussion

This mortality cohort study using a general adult population sample has two main findings. First, more than 70% of the deceased persons had one or more alcohol- or tobacco-attributable disorders. Second, risky alcohol drinking and daily tobacco smoking at baseline were related with time to death as expected according to the combinations of alcohol- and of tobacco-attributable health disorders if all diagnoses including the established but not the suggestive tobacco-attributable in the death certificates were considered.

### Prevalence of attributable disorders

The proportions of deaths that are attributable to alcohol drinking or tobacco smoking among all deaths turned out to be larger than 70%. This finding is in contrast to 12.9% alcohol-attributable deaths according to the National Vital Statistics in the United States of America [31] and 17% (females) to 21% (males) among all deaths at age 50 to 84 in the United States [32]. Four reasons for our high proportions compared to research done before are that we considered all combinations of alcohol- or tobacco-attributable disorders, we considered disorders that are fully and disorders that are partly attributable to alcohol drinking or tobacco smoking, we considered more than one disorder in each of the estimates, and our data are from a general population with particularly high proportions of current daily smokers and of alcohol drinkers at baseline.

First, the data speak in favor of estimating all four combinations of alcohol- or tobacco-attributable disorders. Considering tobacco smoking only would apparently be confounded by alcohol consumption and vice versa. Second, in a part of studies about alcohol-attributable death the fully attributable disorders only were analyzed [33]. However, the difference between fully and partly alcohol-attributable disorders is just that the fully attributable would not exist without alcohol consumption whereas partly alcohol-attributable disorders may also be present in people who do not drink alcohol. In both conditions, alcohol consumption may have the same strong causation of death or disease. Our data revealed relations between risky alcohol consumption and time to death among the decedents with a fully attributable diagnosis in the death certificate and the decedents with a partly attributable disorder as well. The particularly high SHR among the decedents with a fully attributable disorder may be due to the fact that they included a particularly high proportion of harmful drinkers in addition to the high proportion of daily tobacco smokers at baseline. However, it has to be considered that we do not have criteria for high SHR. Third, in the progress of epidemiological research, the number of attributable disorders increased. If all disorders including suggestive tobacco-attributable are considered more than 99% of the death certificates disclosed an attributable disorder. This finding speaks in favor of using total instead of attributable mortality whenever the risk of drinking alcohol or smoking tobacco is to be presented in the practice of public health, particularly if potential effects of changes in alcohol or tobacco consumption in a general population are to be estimated. Two or more disorders were considered even if underlying causes of death only were analyzed. It is of debate whether one or more underlying causes of death only or whether the full spectrum of information available from a death certificate should be used. One reason not to rely on one underlying cause of death is that a variety of faults in death certificates have been reported [11–15]. The required information for the death certificate may not be available for the certifying physician in each death. One of the most severe mistakes according to the underlying cause of death has been a wrong chain of disorders that led to death. Limiting the analysis to underlying causes of death would omit other disorders that might contribute to death. Among all decedents, 28.4% had an underlying cause of death and 51.8% any disorder attributable both to alcohol at-risk drinking and tobacco smoking if only established tobacco-attributable disorders are considered. Using all disorders confirms approaches proposed before [18–20]. Fully alcohol- or tobacco-attributable disorders are underestimated by the underlying cause of death. It failed to detect 7.5% of all deaths as fully attributable in the present study. This speaks in favor of analyzing more than one diagnosis in the death certificate. Also, the assumption that all disorders may constitute a network of causal factors seems to be plausible. Fifth, our data are from a general population with high alcohol consumption [34] and tobacco smoking. Among our sample with vital statistics data, 39.5% were current smokers at baseline [35].

### Risky alcohol consumption or tobacco smoking at baseline and combinations of attributable disorders at mortality follow-up

The results speak in favor of the attributable disorder combinations being valid. The four assumptions are fulfilled by the findings about all disorders mentioned in the death certificate including established but not suggestive tobacco-attributable disorders. Among the deceased persons with fully attributable and among those with partly alcohol- and tobacco-attributable disorders in the death certificate, risky alcohol drinking and daily tobacco smoking at baseline were related to a decreased time to death. Among the deceased with only tobacco-attributable disorders in the death certificate, only daily smoking but not risky alcohol drinking at baseline, and among those with only alcohol-attributable disorders in the death certificate, only alcohol at-risk drinking but not tobacco smoking at baseline was related to an increased likelihood of a short time to death. Among decedents who had neither an alcohol- nor a tobacco-attributable condition in their death certificate, both alcohol at-risk consumption and daily smoking were unrelated to a decreased time to death. The findings support the combinations of alcohol- and tobacco-attributable disorders. They speak in favor of using the four combinations if mortality risks in relation to alcohol consumption or tobacco smoking are to be estimated. It should be kept in mind that the death certificates are no data gathering instruments of research and that multiple error has been revealed in studies done before [11–15]. These preconditions add to the findings of this study being persuasive.

The data do not confirm tobacco smoking to be more important than alcohol at-risk drinking in the prediction of time to death which might be concluded from a higher prevalence of tobacco smoking [31] compared to risky alcohol consumption [32]. Among all deaths in our study, 65.1% are partly attributable to tobacco smoking and 63.7% are partly attributable to alcohol at-risk drinking if all disorders including established but not suggestive tobacco-attributable disorders are considered. This was found although the disorders that were partly attributable to alcohol only were derived from the remaining deaths after fully attributable, partly attributable to alcohol and tobacco and partly attributable to tobacco only had been excluded. In addition, more than 70% of the fully attributable deaths were attributable to alcohol consumption.

## Strengths and limitations

Strengths of our study include that a general population sample with interview data about alcohol and tobacco consumption at baseline and deaths over twenty years were used. This provided findings about the real world of death certificate information in a random adult general population sample. Limitations of this study include that the number of deaths was not sufficient for all data analyses. The cohort study design precludes causal relations. Further health risk factors such as poor diet and low exercise were not considered. The interview data may be biased by reporting error. Particularly alcohol consumption is likely to be underreported. We have no data about changes in alcohol consumption or tobacco smoking from the time between baseline and mortality follow-up. The death certificate data are likely to be biased. Certifying physicians may have had insufficient information about the deceased persons. It may depend on the single physician how much information s/he is open to provide in the death certificate. Memory deficits may have been active. Error in a decision for a health disorder is possible. For some study participants vital status or death certificate data is missing.

## Conclusions

First, more than 70% of the deceased persons had one or more alcohol- or tobacco-attributable disorder. This finding suggests that total mortality seems to be the suitable outcome if potential effects of alcohol or tobacco consumption in a general population are to be estimated. Second, risky alcohol drinking and daily tobacco smoking at baseline were related to time to death as expected according to the combinations of alcohol- and of tobacco-attributable health disorders if all diagnoses including the established but not the suggestive tobacco-attributable in the death certificates were considered. The findings speak in favor of using both alcohol consumption and tobacco smoking if attributable deaths are to be estimated.

## Electronic supplementary material

Below is the link to the electronic supplementary material.

Supplementary Material 1

## Data Availability

The datasets used during the current study are available from the corresponding author on reasonable request.

## Abbreviations

CI: 95% confidence interval
SHR: Subhazard ratio
