# Supplementary material for: Estimating mortality attributable to alcohol or tobacco – a cohort study from Germany
Source: Subst Abuse Treat Prev Policy. 2025 Jan 22;20:5. doi: 10.1186/s13011-025-00633-1 (PMC11755885; doi:10.1186/s13011-025-00633-1)
Supplement: Supplementary file 1 — Supplementary Material 1 [file 13011_2025_633_MOESM1_ESM.docx]

**Additional File 1**

Attributable disorders that have been used in the data analysis

| **Disorder** |  | **ICD-10 code** |
| --- | --- | --- |
| **Fully attributable** |  |  |
| Mental or behavioral disorder due to alcohol use |  | F10 |
| Mental or behavioral disorder due to psychoactive substances other than alcohol or nicotine |  | F11-F16, F18-F19 |
| Mental or behavioral disorder due to tobacco use |  | F17.1-F17.3, F17.8, F17.9 |
| Nervous system degeneration due to alcohol |  | G31.2 |
| Polyneuropathy alcoholic |  | G62.1 |
| Myopathy alcoholic |  | G72.1 |
| Cardiomyopathy alcoholic |  | I42.6* |
| Gastritis alcoholic |  | K29.2** |
| Liver disease, alcoholic |  | K70** |
| Pancreatitis acute, alcohol-induced |  | K85.2** |
| Pancreatitis chronic, alcohol-induced |  | K86.0** |
| Poisoning by and exposure to alcohol |  | X45, Y15 |
| Self-poisoning by and exposure to alcohol, intentional |  | X65 |
|  |  |  |
| **Partly attributable to alcohol and tobacco, established** |  |  |
| Cancer tongue, oral cavity, tonsil, pharynx |  | C01-C06, C09-C10, C12-C14 |
| Cancer oesophagus |  | C15 |
| Cancer stomach |  | C16 |
| Cancer colon, rectum |  | C18, C20 |
| Cancer liver |  | C22 |
| Cancer pancreas |  | C25 |
| Cancer larynx |  | C32 |
| Heart disease, ischemic |  | I20-I25 |
| Atrial fibrillation, flutter |  | I48 |
| Intracranial bleeding |  | I60-I62 |
| Cerebrovascular diseases |  | I63, I65-I67 |
| Sequelae of cerebrovascular disease |  | I69.0- I69.3 |
| Pneumonia |  | J12-J16, J18 |
|  |  |  |
| **Partly attributable to alcohol and tobacco, suggestive** |  |  |
| Cancer breast (women) |  | C50 |
| Cancer prostate |  | C61 |
| Hypertensive diseases |  | I10, I11, I15 |
| Gastro-oesophageal laceration-haemorrhage syndrome |  | K22.6 |
| Liver cirrhosis |  | K74.0-K74.2, K74.6 |
| Gallbladder, biliary tract disease |  | K80, K81, K83 |
| Pancreatitis acute |  | K85.0, K85.1, K85.3-K85.9 |
| Pancreatitis chronic |  | K86.1 |
| Convulsions, other or unspecified |  | R56.8 |
|  |  |  |
| **Partly attributable to tobacco only, established** |  |  |
| Tuberculosis |  | A16-A19 |
| Cancer lips, oral cavity, nasopharynx |  | C00, C07, C08, C11 |
| Cancer rectosygmoid |  | C19 |
| Cancer trachea, bronchus, lung |  | C33-C34 |
| Cancer cervix uteri, collum |  | C53 |
| Cancer kidney, renal pelvis |  | C64-C66 |
| Cancer urinary bladder |  | C67 |
| Leukemia acute myeloid |  | C92.0 |
| Diabetes mellitus |  | E10-E14 |
| Rheumatic fever, rheumatic heart diseases, diseases of pulmonary circulation, heart diseases other |  | I00-I09, I26-I42.5, I42.7-I47, I49-I51 |
| Stroke |  | I64 |
| Cerebrovascular disorders in diseases classified elsewhere |  | I68 |
| Sequelae of cerebrovascular diseases |  | I69.4, I69.8 |
| Diseases of arteries |  | I70-I78 |
| Influenza |  | J10-J11 |
| Pneumonia in diseases classified elswhere |  | J17 |
| Chronic obstructive pulmonary disease |  | J40-J44 |
|  |  |  |
| **Partly attributable to tobacco only, suggestive** |  |  |
| Infections |  | A00-A15, A20-B99 |
| Cancer, rare |  | C17, C21, C23, C24, C26, C30, C31, C37-C41, C45-C49, C51, C52, C58, C60, C62, C63, C68-C70.1, C72-C79, C81.0-C81.3, C96, C97, D00-D48.7 |
| Cancer uterus |  | C54, C55 |
| Cancer, site unspecified |  | C80, D48.9 |
| Disorder eyes |  | H00-H59 |
| Disorder ears |  | H60-H95 |
| Disorder respiratory system |  | J00-J09, J20-J39, J45-J84.0, J84.8-J99 |
| Disorder digestive system |  | K00-K22.5, K22.7-K29.1, K29.3-K52, K56-K67, K82, K84, K86.2-K93 |
| Vascular disorder intestinal system |  | K55 |
| Liver cirrhosis |  | K74.3-K74.5 |
| Disorder skin |  | L00-L99 |
| Musculoskeletal disorder |  | M00-M99 |
| Kidney failure, disease |  | N17-N19 |
| Disorders pregnancy, childbirth |  | O00-O99 |
| Disorder originating in perinatal period |  | P00-P96 |
| Congenital malformations |  | Q00-Q99 |
| Symptoms, signs not specified elsewhere |  | R00-R56.0, R57-R96 |
| Disorder unspecified |  | R99 |
|  |  |  |
| **Partly attributable to alcohol only** |  |  |
| Epilepsy, status epilepticus, convulsions |  | G40, G41 |
| Cerebral ischaemic attacks, transient |  | G45 |
| Hypertensive renal disease |  | I12, I13 |
| Varices oesophageal |  | I85 |
| Hepatitis, chronic |  | K73 |
| Liver diseases other, not alcohol |  | K76.0, K76.6, K76.7, K76.9 |
| Accident transport land |  | V01-V89 |
| Accident transport water |  | V90-V94 |
| Accident transport air |  | V95-V97 |
| Fall |  | W00-W19, Y30 |
| Contact with sharp objects, machinery, foreign objects |  | W24-W31, W45 |
| Firearm discharge |  | W32-W34, Y22-Y24 |
| Drowning |  | W65-W70, W73, W74, Y21 |
| Inhalation causing obstruction of respiratory tract |  | W78-W79 |
| Exposure to smoke, fire, flames |  | X00-X06, X08, X09, Y26 |
| Exposure to excessive natural cold |  | X31 |
| Poisoning, not alcohol |  | X40-X44, X46-X49, Y10-Y14, Y16-Y19 |
| Self-harm intentional |  | X60-X64, X66-X84, Y87.0 |
| Assault |  | X85-X99, Y00-Y09, Y87.1 |

* Additionally attributable to tobacco, established.

** Additionally attributable to tobacco, suggestive.
